# Dietary protein intake, inflammatory biomarkers, genetic susceptibility, and the incidence of sarcopenia: a prospective population-based study

**DOI:** 10.3389/fnut.2026.1821758

**Published:** 2026-05-04

**Authors:** Hongxia Xia, Rong Xiang, Xin Song, Yang Qu, Xunying Zhao, Ting Liu, Maoyao Xia, Yangdan Zhong, Zilan Chen, Ye Ju, Yuqi Pang, Zihao Li, Mengyu Fan, Lu Long, Xia Jiang

**Affiliations:** 1 Department of Epidemiology and Biostatistics, West China School of Public Health and West China Fourth Hospital, Sichuan University, Chengdu, Sichuan, China; 2 Department of Nutrition and Food Hygiene, West China School of Public Health and West China Fourth Hospital, Sichuan University, Chengdu, Sichuan, China; 3 Department of Clinical Neuroscience, Center for Molecular Medicine, Karolinska Institutet, Stockholm, Sweden

**Keywords:** dietary protein intake, genetic susceptibility, inflammation biomarkers, prospective cohort study, sarcopenia

## Abstract

**Background:**

Sarcopenia elevates risks of falls, hospitalization, and mortality in older adults. Despite protein’s importance for muscle maintenance, evidence on its overall and source-specific role in sarcopenia development remains inconclusive, particularly regarding interactions with inflammation and genetic factors.

**Methods:**

We analyzed data from 37,870 participants in the UK Biobank. Cox proportional hazard models were used to evaluate the association between dietary protein intake and incident sarcopenia. Mediation analyses were conducted to assess whether inflammatory biomarkers mediate the dietary protein intake-sarcopenia association. A polygenic risk score (MetaPRS) for sarcopenia was constructed to explore the joint and interactive effects of dietary protein intake and genetic susceptibility on sarcopenia risk.

**Results:**

Higher dietary plant protein intake, but not total or animal protein, was modestly associated with a lower incidence of sarcopenia. Specifically, individuals in the highest intake quartile had a 25% lower risk than those in the lowest (HR = 0.75, 95%CI = 0.58–0.98) in the fully-adjusted model. Mediation analysis suggested that CRP, WBC count, monocyte count, and platelet count to explain 9.9% (95%CI = 4.8–41.5%) of the association, indicating that inflammatory biomarkers may partly contribute to this association.

**Conclusion:**

Higher dietary plant protein intake is modestly associated with a lower incidence of sarcopenia, and inflammatory biomarkers may partly contribute to this association.

## Introduction

Sarcopenia is a progressive degenerative disorder characterized by significant declines in skeletal muscle mass and function (1, 2). It predominantly affects older adults and is associated with an increased risk of subsequent falls, hospitalization, and mortality (3, 4). In the absence of effective pharmacological treatments, the existing prevention strategies primarily focus on modifiable lifestyle factors (1). Dietary protein intake has emerged as a key nutritional exposure and modifiable determinant of muscle health, owing to its essential role in stimulating muscle protein synthesis (MPS) and preserving lean mass (5).

Current epidemiological evidence regarding the association of dietary protein intake with the risk of sarcopenia remains inconsistent. One small-scale meta-analysis of four cross-sectional studies involving a total of 768 older adults found a significantly lower protein intake among those with sarcopenia as compared to sarcopenia-free individuals [standardized mean difference (SMD) = 0.37, 95%CI = 0.19–0.55, *p* < 0.001] (6). This was not supported by the longitudinal Newcastle 85 + study involving 757 older adults, which suggested higher sarcopenia incidence over 3 years among those consuming ≥ 1 g/kg body weight per day of protein (OR = 2.57, 95%CI = 1.26–5.26, *p* = 0.01) (7). On the contrary, another prospective study involving 3,380 community-dwelling Chinese older adults reported no significant association (8). Notably, most current existing epidemiological studies related to this topic were of cross-sectional design, with limited sample size, and lacked adequate adjustment of key confounders such as physical activity and comorbidities, calling for a well-designed, large-scale longitudinal study to address these limitations and to better clarify the role of dietary protein intake in the onset of sarcopenia.

The source of dietary protein-typically categorized as animal- or plant-based-differentially influences muscle health. Animal-based proteins in general stimulate MPS more effectively due to higher digestibility and superior essential amino acid profiles (9, 10), though some sources may contain potentially pro-inflammatory compounds (11). Plant-based proteins, while less efficient at MPS stimulation due to incomplete amino acids and lower leucine bioavailability, contain beneficial bioactive compounds like polyphenols and fibers that support muscle health through anti-inflammatory and antioxidant effects (12, 13). However, epidemiological evidence on the association between dietary protein sources and sarcopenia risk remains inconsistent. Some studies have linked higher plant protein intake to lower sarcopenia risk (8, 14), while others reported protective associations with animal protein (15). More research is needed to elucidate this relationship.

Inflammation has been consistently linked to an accelerated muscle loss, reduced muscle strength, and impaired function in older adults, therefore serving as an important biological mechanism underlying sarcopenia (16, 17). Emerging evidence has suggested that dietary protein intake mitigates inflammation and oxidative stress (18), offering protection against inflammation-induced sarcopenia. However, only one study so far has explicitly examined the interaction between dietary protein intake and inflammatory biomarkers with sarcopenia and confirmed a potential synergistic protective effect between plant protein intake and levels of C-reactive protein in a cohort of 569 sarcopenic Chinese older adults (*P*_interaction_ = 0.048) (8). Whether additional inflammatory mechanisms play a role in the dietary protein–sarcopenia relationship remains unknown.

Genetic susceptibility plays a critical role in the pathogenesis of sarcopenia and may be modified by environmental exposures. For example, a higher polygenic risk score (PRS) of sarcopenia has been shown to amplify the adverse effect of air pollution on disease prevalence (19). Similarly, dietary protein intake has been reported to attenuate the genetic risk in conditions such as obesity (20). However, whether dietary protein intake modulates genetic susceptibility to sarcopenia remains unexplored.

Therefore, leveraging data from the large-scale prospective population-based UK Biobank cohort, we aimed to (1) evaluate the associations of total, animal-, and plant-based dietary protein intake with the risk of incident sarcopenia; (2) assess whether inflammatory biomarkers mediate the dietary protein-sarcopenia associations; and (3) explore the joint and interactive effects of dietary protein intake and genetic susceptibility on sarcopenia risk.

## Materials and methods

### Study population

We used data from the UK Biobank, an ongoing prospective cohort comprising over 500,000 participants aged 37–73 years at baseline, recruited between 2006 and 2010 (accessed under Application no. 99713). Participants provided biological samples, completed touchscreen questionnaires, and underwent physical assessments. Detailed protocols have been reported previously (21). All participants gave written informed consent, and ethical approval for the UK Biobank was granted by the North West Multi-centre Research Ethics Committee. Because the dataset provided to researchers is fully deidentified, no additional ethical approval was required for the present study.

**Figure 1:**
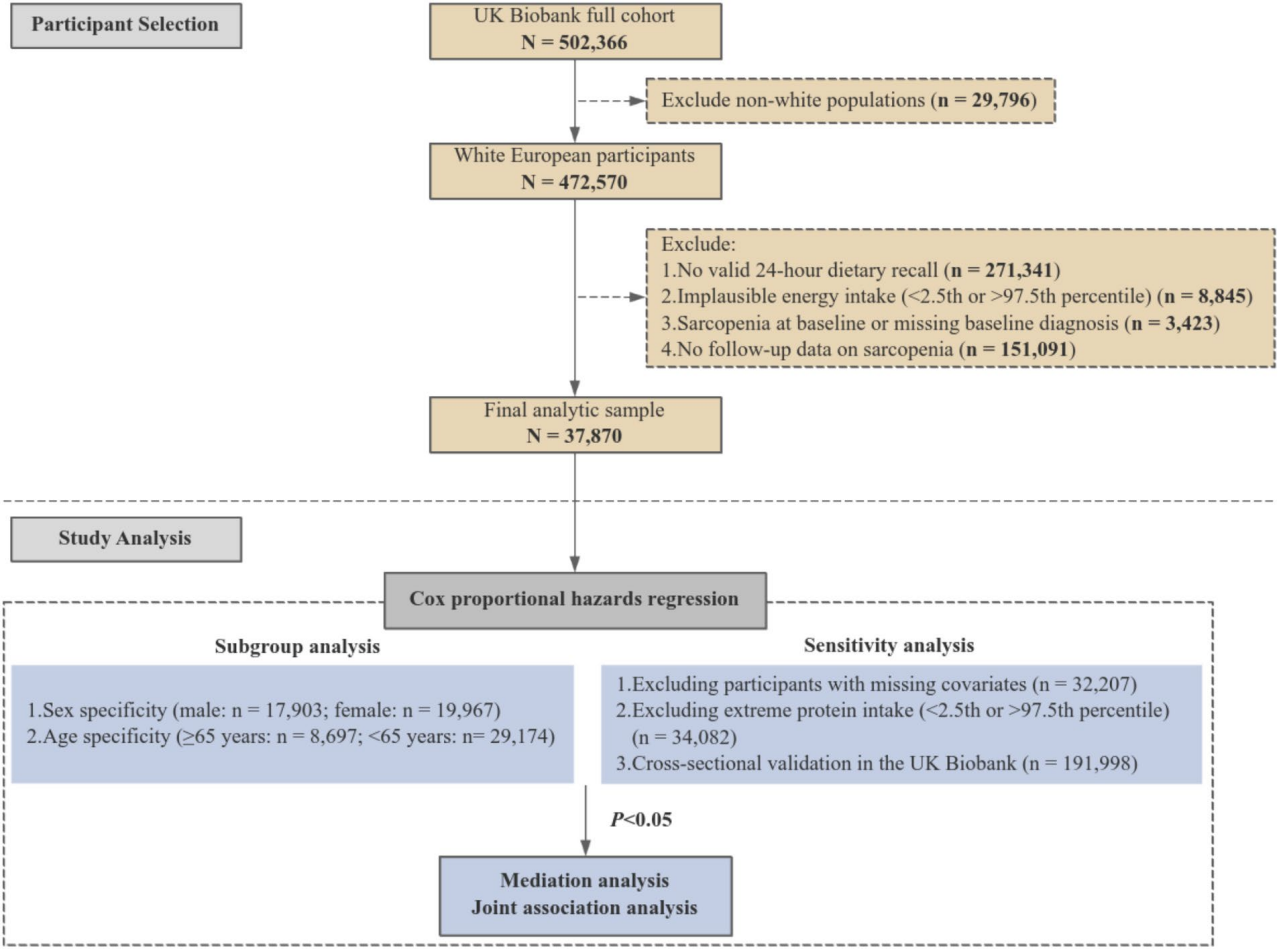
Flowchart of participant selection and study design.

In this study, we initially identified 472,570 white European participants. Subsequent exclusion criteria included (i) no valid 24-h dietary recall (*n* = 271,341); (ii) implausible energy intake (< 2.5th or > 97.5th percentile; *n* = 8,845); (iii) a diagnosis of sarcopenia at baseline or missing diagnostic data (*n* = 3,423), and (iv) no follow-up information on sarcopenia (*n* = 151,091). Finally, a total of 37,870 participants were included in the final analysis (Figure 1).

### Dietary protein assessment

Dietary nutrient intake was assessed using the Oxford WebQ, a web-based 24-h dietary recall questionnaire covering 206 foods and 32 beverages, up to five occasions between April 2009 and June 2012 (22–24). Total protein intake was derived from twelve predefined food categories-whole grains, nuts, legumes, red meat, processed meat, poultry, egg and egg dishes, oily fish, non-oily fish, cheese, milk, and yogurt-as described in Supplementary Table 1. Each reported food was linked to standardized portion sizes based on the UK reference Food Portion Sizes (2nd edition, 1993) and nutrient composition data from the UK Nutrient Databank (UKNDB, 2013). Protein intake was calculated by multiplying the consumed quantity of each food item by its protein content per gram, as provided by the UKNDB, and then summing the protein contributions from all reported foods. The total protein intake was subsequently categorized into animal and plant protein based on the origin of the food items (24).

For each participant, the average daily intake of total protein (Field ID 26005, g/day), plant protein (Field ID 26006, g/day), and animal protein (Field ID 26007, g/day) was calculated as the mean across all five dietary assessments. Protein intake was then categorized into quartiles (the lowest Q1 to the highest Q4) based on its distribution within the study population.

### Assessment of sarcopenia

Sarcopenia was defined according to the 2019 European Working Group on Sarcopenia in Older People (EWGSOP2) criteria, characterized by low muscle strength and low muscle mass, with or without impaired physical performance (25). As the UK Biobank does not follow standard clinical diagnostic protocols (26), we defined sarcopenia in this study as the coexistence of (1) low grip strength, defined as < 27 kg for men and < 16 kg for women; and (2) low muscle mass, defined as appendicular lean soft tissue (ALST) divided by body mass index (BMI), with thresholds of ALST/BMI < 0.84 in men and < 0.55 in women. The method for estimating ALST has been described previously (27).

Incident sarcopenia was identified using repeated grip strength and body composition measurements collected across up to four follow-up assessment visits. Participants who met the diagnostic criteria at baseline were excluded, and incident sarcopenia was defined as the first follow-up visit at which both low grip strength and low muscle mass criteria were simultaneously satisfied, with the event date assigned as the date of that visit (Field ID 53). Person-years of follow-up were calculated from the date of the first completed 24-h dietary recall to the earliest of incident sarcopenia, death, loss to follow-up, or end of follow-up. Participants who remained free of sarcopenia were censored at their last available assessment date.

### Assessment of inflammatory biomarkers

To investigate the mediating role of inflammation, we chose biomarkers reflecting different inflammatory processes: lymphocyte count (Field ID 30120, 10^9^ cells/L), white blood cell (WBC) count (Field ID 30000, 10^9^ cells/L), platelet count (Field ID 30080, 10^9^ cells/L), neutrophil count (Field ID 30140, 10^9^ cells/L), and C-reactive protein level (Field ID 30710, mg/L).

### Construction of MetaPRS

The MetaPRS for sarcopenia, integrating polygenic risk for muscle mass and handgrip strength, was constructed via three stages. First, we extracted independent single nucleotide polymorphisms (SNPs) that showed significant genome-wide association with muscle mass (28) and grip strength (29) in recently published genome-wide association studies (GWASs). Then, we used summarized SNP data for muscle mass and handgrip strength to generate a range of scores based on different r^2^ thresholds with PLINK LD thinning (− indep-pairwise) and selected the best model (in terms of the largest magnitude hazard ratio), resulting in one representative trait-specific PRS for each trait (muscle mass and handgrip strength). After that, each trait-specific PRS was standardized to a zero mean and unit standard deviation over the entire dataset. Next, we employed elastic-net logistic regression (30) using the R package ‘glmnet’ (31) to model the associations between the two trait-specific PRSs and sarcopenia. A grid of models spanning different penalty strengths was evaluated via 10-fold cross-validation. The model demonstrating the highest cross-validated area under the receiver operating characteristic curve (AUC) was selected and used. Subsequently, the sarcopenia-associated MetaPRS was derived as a weighted linear combination of the standardized trait-specific PRSs, using coefficients estimated from the final elastic-net models.

### Assessment of covariates

Covariates included age (continuous, years), sex (male or female), education level (university degree or other), Townsend Deprivation Index (TDI, continuous), body mass index (BMI, <25, 25–30 or ≥ 30 kg/m^2^), total physical activity level (low, moderate, or high activity group), smoking status (never, previous, or current), alcohol consumption (never, previous, or current), hypertension status (yes or no), hyperlipidemia status (yes or no), diabetes status (yes or no), and energy intake (continuous, kcal/day). TDI was an area-based index, with a lower value indicating a higher socioeconomic status.

### Statistical analysis

Baseline characteristics were described as means (SD) for continuous variables and numbers (%) for categorical variables, and differences across quartiles of total dietary protein intake were tested using one-way ANOVA for continuous variables and chi-square test for categorical variables. For missing data, continuous variables were imputed using the median and missing categories for categorical variables were treated as a separate category.

Cox proportional hazard models were employed to examine the associations between dietary protein intake and incident sarcopenia. The Schoenfeld residual plots were applied to confirm that the Cox models satisfied the proportional hazards assumption. Hazard ratios (HRs) with corresponding 95% confidence intervals (CIs) were evaluated in three models: (1) model 1 (basic model): adjusted for age and sex; (2) model 2: additionally adjusted for education level, Townsend deprivation index, body mass index, total physical activity level, smoking status, and alcohol consumption on top of model 1; (3) model 3 (full model): additionally adjusted for hypertension, hyperlipidemia, diabetes, and energy intake on top of model 2. Restricted cubic splines (RCS) were used to investigate the dose–response association.

Stratified analyses were performed to assess the potential modifiable effects, including age and sex. Interaction was assessed by adding a multiplicative term in the full model and tested using the likelihood ratio method, comparing models with and without the term. To assess the robustness of our findings, we conducted several sensitivity analyses: (1) excluding participants with missing covariates; (2) excluding those with extreme dietary protein intake (<2.5th or >97.5th percentile); (3) conducting a cross-sectional validation in the UK Biobank; and (4) fitting logistic regression models that do not rely on the exact timing of event occurrence, with incident sarcopenia defined as a binary outcome over the follow-up period.

Mediation analyses [using the R “CMAverse” package (32)] were performed to test whether the associations between dietary protein intake (exposure) and sarcopenia incidence (outcome) were mediated by inflammatory biomarkers (mediator). The proportion mediated (PM) was quantified during this process. Linear regression models were fitted for the exposure-mediator models, while Cox proportional hazards models were fitted for the exposure-outcome and mediator-outcome models. We further examined the joint and interactive effects of dietary protein intake and genetic susceptibility on sarcopenia risk, with the reference group being the lowest-risk participants.

R version 4.4.2 was used for data analysis. A two-sided *p*-value < 0.05 was considered statistically significant.

## Results

### Study population characteristics

Over a median follow-up of 11.45 years, 819 incident sarcopenia cases were identified among 37,870 participants. Baseline characteristics across quartiles of total dietary protein intake are presented in Table 1. Of all participants, 52.73% were females, and the mean age was 58.20 years (SD = 7.58). Compared with those in the lowest quartile, individuals in the highest quartile of total dietary protein intake were more likely to be older, male, better educated, and of higher socioeconomic status. They also had higher BMIs, lower prevalence of current smoking, more baseline comorbidities, and greater overall nutrient intake.

**Table 1:** Characteristics of participants across quartiles of total dietary protein intake.

Characteristics	Overall (*N* = 37,870)	Quartiles of Total Dietary Protein Intake (g/day)				
		Q1	Q2	Q3	Q4	*p*-value
Basic characteristics						
Age, years	58.20 ± 7.58	57.81 ± 7.62	58.52 ± 7.49	58.49 ± 7.44	58.01 ± 7.74	<0.001
Sex						<0.001
Female	19,967 (52.73)	5,961 (62.95)	5,458 (57.66)	4,827 (50.99)	3,721 (40.46)	
Male	17,903 (47.27)	3,508 (37.05)	4,008 (42.34)	4,640 (49.01)	5,475 (59.54)	
College or University Degree	24,797 (65.48)	6,070 (64.10)	6,195 (65.44)	6,333 (66.90)	6,199 (65.47)	<0.001
Body mass index (kg/m^2^)	26.48 ± 4.22	26.08 ± 4.17	26.18 ± 4.14	26.50 ± 4.12	27.16 ± 4.38	<0.001
Townsend deprivation index	−2.02 ± 2.64	−1.82 ± 2.76	−2.10 ± 2.60	−2.11 ± 2.55	−2.04 ± 2.64	<0.001
High physical activity level	12,587 (33.24)	2,988 (31.56)	3,097 (37.93)	3,179 (33.58)	3,323 (35.10)	0.002
Sleep 7–8 h/day	27,734 (73.23)	6,765 (71.44)	7,008 (74.03)	7,068 (74.66)	6,893 (72.80)	<0.001
Smoking status						<0.001
Never	22,832 (60.30)	5,692 (60.11)	5,732 (60.55)	5,833 (61.61)	5,575 (58.88)	
Previous	12,839 (33.90)	3,157 (33.34)	3,223 (34.05)	3,168 (33.46)	3,291 (34.76)	
Current	2,130 (5.62)	606 (6.40)	490 (5.18)	451 (4.76)	583 (6.16)	
Missing	69 (0.18)	14 (0.15)	21 (0.22)	15 (0.17)	19 (0.20)	
Drinking status						<0.001
Never	787 (2.08)	239 (2.53)	188 (1.99)	187 (1.96)	173 (1.83)	
Previous	827 (2.18)	254 (2.68)	210 (2.22)	172 (1.81)	191 (2.02)	
Current	36,251 (95.73)	8,975 (94.78)	9,067 (95.78)	9,106 (96.21)	9,103 (96.14)	
Missing	5 (0.01)	1 (0.01)	1 (0.01)	2 (0.02)	1 (0.01)	
Hypertension	2,562 (6.77)	578 (6.10)	616 (6.51)	634 (6.70)	734 (7.75)	<0.001
Hyperlipidemia	1,101 (2.91)	259 (2.74)	261 (2.76)	261 (2.76)	320 (3.38)	0.019
Diabetes	418 (1.10)	92 (0.97)	98 (1.04)	111 (1.17)	117 (1.24)	0.279
Follow-up time (year)	11.45 ± 1.44	11.41 ± 1.47	11.50 ± 1.39	11.47 ± 1.39	11.41 ± 1.49	<0.001
Nutrients intake						
Energy intake (kcal/day)	2046.69 ± 469.83	1648.36 ± 349.70	1921.41 ± 337.60	2148.99 ± 356.83	2468.02 ± 396.25	<0.001
Carbohydrate intake (g/day)	251.67 ± 65.64	214.48 ± 56.15	240.62 ± 55.28	262.96 ± 58.96	288.64 ± 67.48	<0.001
Fat intake (g/day)	72.71 ± 23.96	55.94 ± 18.52	66.90 ± 19.20	76.12 ± 20.37	89.88 ± 23.43	<0.001
Plant protein (g/day)	28.21 ± 9.10	23.68 ± 7.97	26.69 ± 7.73	29.42 ± 8.12	33.06 ± 9.71	<0.001
Animal protein (g/day)	52.15 ± 18.99	32.13 ± 10.37	46.42 ± 8.24	56.07 ± 8.68	73.99 ± 16.29	<0.001
Inflammatory biomarkers*						
CRP (mg/L)	0.12 ± 1.03	0.09 ± 1.05	0.10 ± 1.03	0.13 ± 1.03	0.15 ± 1.00	<0.001
WBC (10^9 cells/L)	1.85 ± 0.25	1.85 ± 0.25	1.85 ± 0.24	1.85 ± 0.24	1.86 ± 0.24	0.002
Lymphocyte count (10^9 cells/L)	0.59 ± 0.31	0.59 ± 0.32	0.59 ± 0.30	0.59 ± 0.31	0.59 ± 0.31	0.781
Monocyte count (10^9 cells/L)	−0.83 ± 0.36	−0.86 ± 0.37	−0.85 ± 0.36	−0.82 ± 0.35	−0.80 ± 0.35	<0.001
Neutrophil count (10^9 cells/L)	1.34 ± 0.32	1.33 ± 0.33	1.34 ± 0.32	1.34 ± 0.32	1.35 ± 0.32	0.021
Platelet count (10^9 cells/L)	5.49 ± 0.23	5.51 ± 0.23	5.50 ± 0.23	5.48 ± 0.23	5.47 ± 0.24	<0.001

Data are presented as mean±standard deviation or number (%). WBC, white blood cell; CRP, C-reactive protein; Q, quartile. *The levels of inflammatory biomarkers were natural log-transformed before analysis.

### Association between dietary protein intake and sarcopenia incidence

As presented in Table 2, no significant association was found between total dietary protein intake and sarcopenia incidence across all three models (model 1: HR = 1.14, 95%CI = 0.95–1.38; model 2: HR = 0.88, 95%CI = 0.71–1.08; model 3: HR = 0.95, 95%CI = 0.72–1.26). However, for plant protein intake, a modestly protective effect was observed. Specifically, in the basic model, individuals in the highest quartile showed a 30% lower risk of sarcopenia than those in the lowest quartile (HR = 0.70, 95%CI = 0.57–0.85). Such a finding remained consistent when adjusting for lifestyle factors (HR = 0.75, 95%CI = 0.60–0.94) and in the full model (HR = 0.75, 95%CI = 0.58–0.98). Notably, a significant dose–response trend was also observed (*P*_trend_ < 0.05). RCS analysis revealed a significant non-linear association (*P*_non-linearity_ = 0.017; Supplementary Figure 1B). For animal protein intake, a positive association was observed only in the basic model (HR = 1.39, 95%CI = 1.14–1.69), of which effect attenuated and became non-significant after additional adjustment (HR = 1.03, 95%CI = 0.82–1.28) or in the full model (HR = 1.12, 95%CI = 0.88–1.43).

**Table 2:** Associations between dietary protein intake and incident sarcopenia.

Protein sources	Protein intake (g/day)	Model 1		Model 2		Model 3 (main model)	
		HR (95%CI)	*p*-value	HR (95%CI)	*p*-value	HR (95%CI)	*p*-value
Total protein							
Q1, lowest	≤66.72	1.00 [Ref]		1.00 [Ref]		1.00 [Ref]	
Q2	66.72–79.13	0.87 (0.72–1.05)	0.148	0.84 (0.68–1.04)	0.108	0.87 (0.69–1.08)	0.206
Q3	79.13–92.60	0.90 (0.74–1.09)	0.275	0.82 (0.66–1.02)	0.078	0.87 (0.68–1.11)	0.253
Q4, highest	≥92.60	1.14 (0.95–1.38)	0.166	0.88 (0.71–1.08)	0.220	0.95 (0.72–1.26)	0.736
Plant protein							
Q1, lowest	≤22.20	1.00 [Ref]		1.00 [Ref]		1.00 [Ref]	
Q2	22.20–27.22	0.73 (0.61–0.88)	**<0.001**	0.80 (0.65–0.98)	**0.032**	0.80 (0.65–0.99)	**0.039**
Q3	27.22–32.96	0.71 (0.59–0.86)	**<0.001**	0.79 (0.64–0.97)	**0.026**	0.79 (0.62–0.99)	**0.045**
Q4, highest	≥32.96	0.70 (0.57–0.85)	**<0.001**	0.75 (0.60–0.94)	**0.011**	0.75 (0.58–0.98)	**0.036**
Animal protein							
Q1, lowest	≤40.17	1.00 [Ref]		1.00 [Ref]		1.00 [Ref]	
Q2	40.17–51.36	1.11 (0.91–1.35)	0.316	1.07 (0.86–1.34)	0.539	1.11 (0.88–1.39)	0.381
Q3	51.36–62.83	1.16 (0.95–1.42)	0.145	1.05 (0.84–1.31)	0.661	1.10 (0.88–1.39)	0.405
Q4, highest	≥62.83	1.39 (1.14–1.69)	**0.001**	1.03 (0.82–1.28)	0.817	1.12 (0.88–1.43)	0.371

HR, Hazard Ratio; CI, confidence interval; Q, quartile. Model 1: adjusted for age and sex. Model 2: additionally adjusted for education level, Townsend deprivation index, body mass index, total physical activity level, smoking status, and alcohol consumption on top of Model 1. Model 3: additionally adjusted for hypertension, hyperlipidemia, diabetes, and energy intake on top of Model 2. Bold values indicate statistical significance (*p* < 0.05).

### Stratified analysis by age and sex

As shown in Supplementary Table 2, in stratified analysis, the inverse association of total dietary protein intake was observed among individuals aged < 65 years (Q4 vs. Q1, HR = 0.79, 95%CI = 0.56–1.13) and males (Q4 vs. Q1, HR = 0.87, 95%CI = 0.52–1.43), although not significant. No multiplicative interaction was observed (all *P*_interaction_ > 0.05). For plant protein intake, the inverse association was observed in both aged ≥ 65 (Q4 vs. Q1, HR = 0.87, 95%CI = 0.57–1.34) and aged < 65 (Q4 vs. Q1, HR = 0.71, 95%CI = 0.50–0.99) individuals as well as in both females (Q4 vs. Q1, HR = 0.73, 95%CI = 0.53–0.99) and males (Q4 vs. Q1, HR = 0.77, 95%CI = 0.49–1.23), although statistical significance was only observed in individuals aged < 65 years and females. Similarly, no multiplicative interaction was observed (all *P*_interaction_ > 0.05). For animal protein intake, no significant association was observed in any subgroup.

### Sensitivity analysis

Results remained robust after excluding participants with missing covariates or extreme protein intake (<2.5th or >97.5th percentile) (Supplementary Table 3) and were further supported by cross-sectional analyses of UK Biobank baseline data (*n* = 191,998) (Supplementary Tables 4, 5). Similar results were observed in logistic regression models that do not rely on the exact timing of events (Supplementary Table 6).

### Mediation analysis

**Figure 2:**
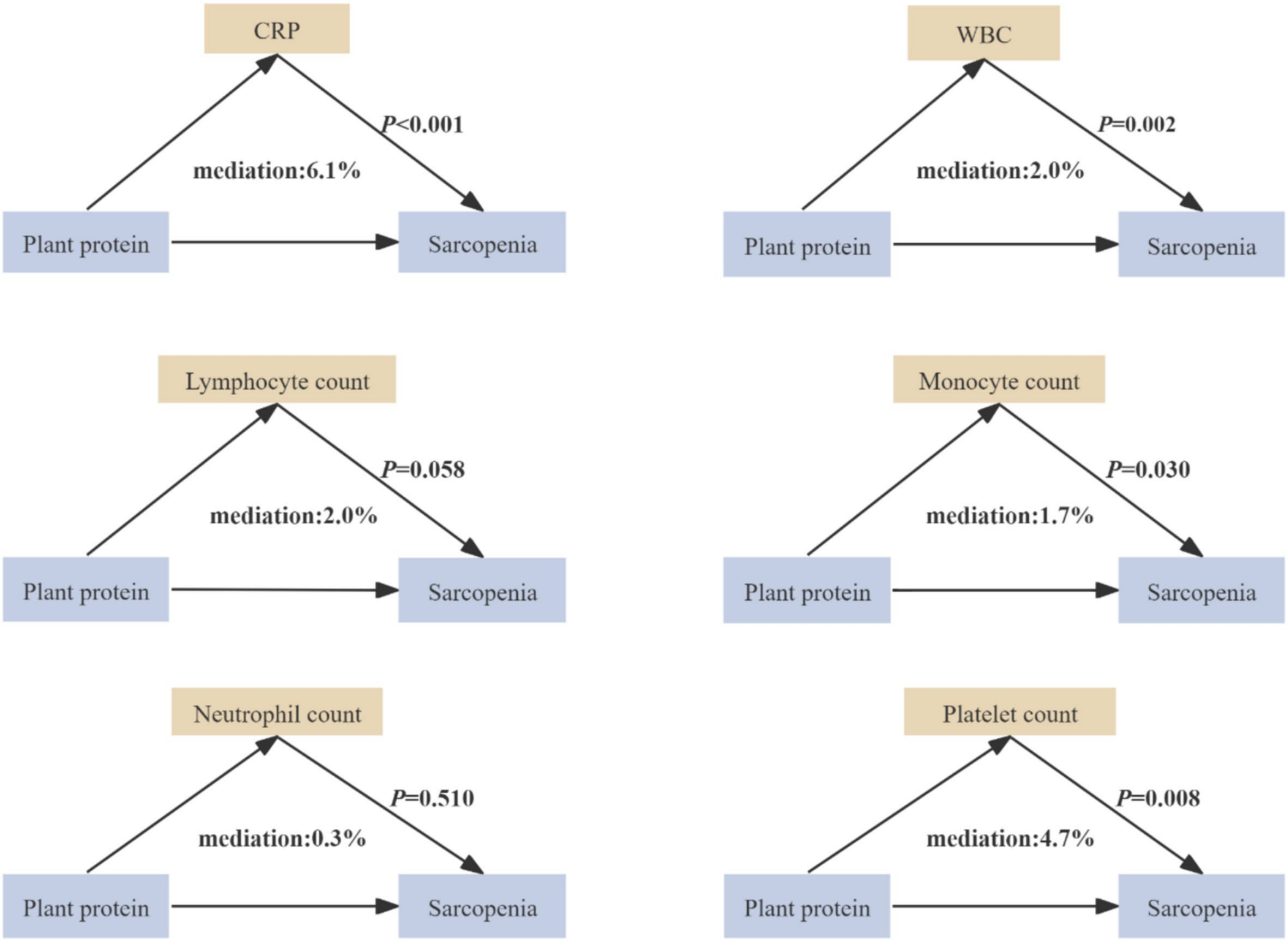
Mediation effects of inflammatory biomarkers on the association between dietary plant protein intake and the incidence of sarcopenia. The mediation analyses were adjusted according to Model 3 covariates: age, sex, education level, Townsend deprivation index, body mass index, total physical activity level, smoking, alcohol consumption, hypertension, hyperlipidemia, diabetes, and energy intake. * *p* < 0.05. WBC, white blood cell; CRP, C-reactive protein. The levels of biomarkers were natural log-transformed before analysis.

Elevated levels of CRP, WBC count, lymphocyte count, monocyte count, neutrophil count, and platelet count were significantly associated with an increased risk of sarcopenia (all *p* < 0.05) (Supplementary Table 7). Mediation analysis showed that CRP (PM = 6.1, 95%CI = 2.5–25.8%), WBC count (PM = 2.0, 95%CI = 0.4–7.6%), monocyte count (PM = 1.7, 95%CI = 0.1–7.4%), and platelet count (PM = 4.7, 95%CI = 1.9–15.3%) each significantly mediated the protective association between plant protein intake and sarcopenia incidence (Figure 2). When including these biomarkers altogether, the combined mediation effect was 9.9% (95%CI = 4.8–41.5%).

### Association of dietary plant protein intake and genetic risk with sarcopenia incidence

**Figure 3:**
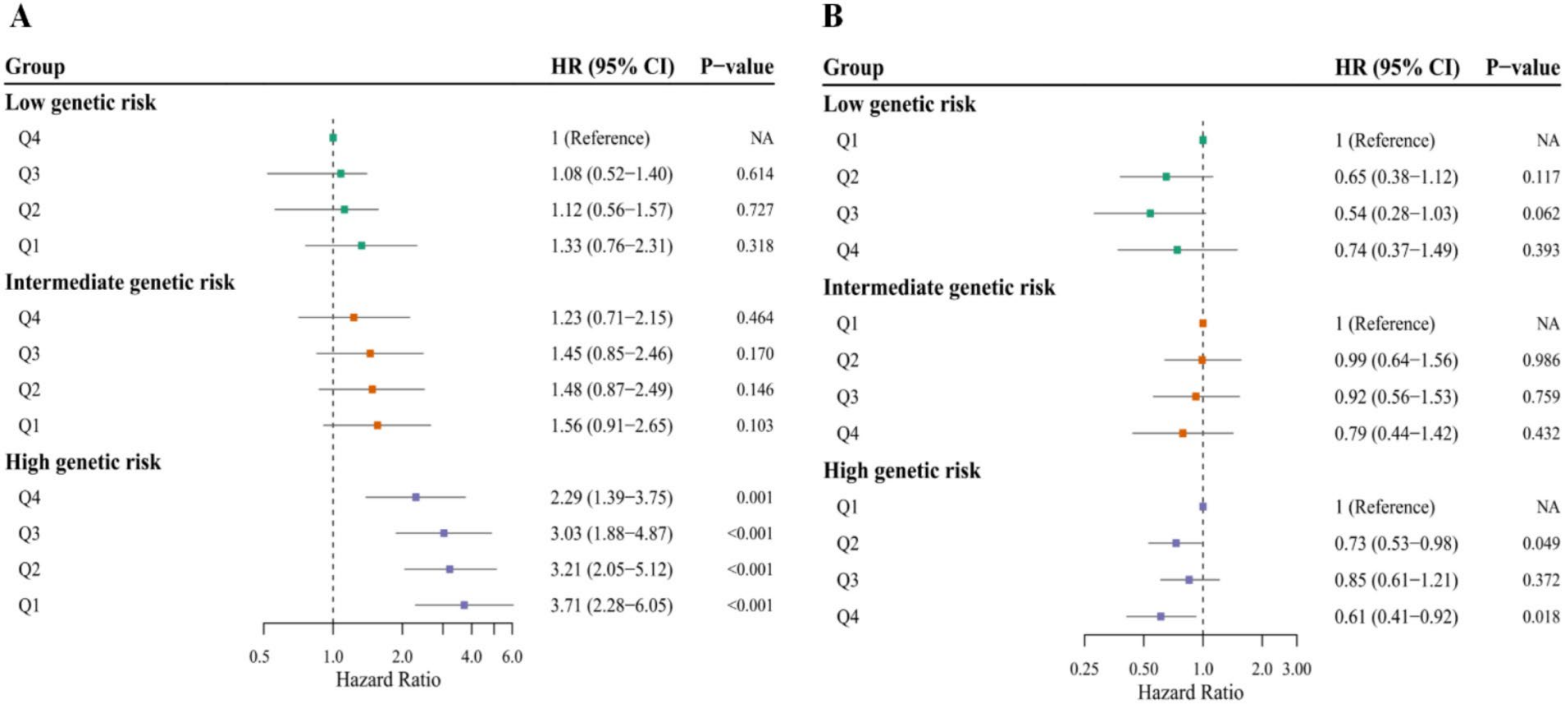
Association of dietary plant protein intake and genetic risk (MetaPRS) with sarcopenia incidence. Panel **(A)** illustrate the joint associations of dietary plant protein intake and genetic risk with sarcopenia incidence. Dietary plant protein was divided into quartiles; genetic risk was evaluated by MetaPRS and categorized into low, intermediate, and high. Individuals with the highest quartile of dietary plant protein intake and low genetic risk were treated as the reference group. Panel **(B)** depicts the stratified analysis of dietary plant protein intake and genetic risk with sarcopenia incidence. Dietary plant protein was divided into quartiles; genetic risk was evaluated by MetaPRS and categorized into low, intermediate, and high. Cox proportional hazards regression analyses were adjusted according to Model 3 covariates: age, sex, education level, Townsend deprivation index, body mass index, total physical activity level, smoking, alcohol consumption, hypertension, hyperlipidemia, diabetes, and energy intake. Q, quartile.

We further evaluated the joint association of dietary plant protein intake and genetic risk (MetaPRS) on sarcopenia incidence (Figure 3A). High genetic risk was strongly associated with sarcopenia incidence (HR = 6.03, 95%CI = 4.62–7.88). Participants with the highest genetic risk and the lowest dietary plant protein intake showed the highest risk of sarcopenia (HR = 3.71, 95%CI = 2.28–6.05). Critically, we observed no evidence of a counteracting effect of higher plant protein intake on the elevated risk conferred by high genetic susceptibility. Stratified analysis (Figure 3B) revealed that higher dietary plant protein intake (Q4 vs. Q1) was associated with a lower risk of sarcopenia (HR = 0.61, 95% CI = 0.41–0.92) among individuals at high genetic risk. However, the multiplicative interaction between genetic risk and plant protein intake regarding sarcopenia risk was not statistically significant (*P*_interaction_ = 0.74).

## Discussion

In this large prospective cohort of middle-aged to older adults, we found that higher dietary plant protein, but not total or animal protein intake, was modestly associated with a lower incidence of sarcopenia. This association remained robust after excluding participants with missing covariates or extreme protein intake and was, in parallel, validated in a larger cross-sectional analysis. Furthermore, results from logistic regression models, which do not rely on the exact timing of events, were similar to those from the primary analysis. Notably, this association may be partly explained by inflammatory biomarkers.

Consistent with prior studies, we found no significant association between total dietary protein intake and sarcopenia incidence in the overall population (8, 33). However, RCS analysis suggested a potential non-linear relationship. Furthermore, in participants aged < 65 years, we observed a mild protective effect when comparing the second quartile of total protein intake to the lowest quartile, with no added benefit in higher quartiles. This pattern may indicate a potential threshold effect, where protein intake above a certain level yields diminishing returns. Indeed, the mean total protein intake of individuals aged < 65 years (1.04 g/kg BW/day) exceeded the current Recommended Dietary Allowance (RDA) of 0.8 g/kg BW/day for adults (34). Since protein intake in this group was adequate for maximal stimulation of muscle protein synthesis, excess protein beyond this threshold is likely oxidized for energy rather than utilized for additional muscle accretion, thereby explaining the lack of additional protective benefit in higher quartiles (35). Conversely, the mean total protein intake of participants aged ≥ 65 years (1.05 g/kg BW/day) was below the recommended intake of 1.2 g/kg BW/day for older adults (34), yet no significant association was observed. This apparent paradox may be primarily explained by age-related anabolic resistance, where in older individuals exhibit a diminished muscle protein synthetic response to amino acids, necessitating consideration of not only higher protein quantity but also protein quality and essential amino acid composition (36). Additionally, the suboptimal protein intake in this age group may have constrained our statistical power to detect a protective effect. Consequently, we propose that current findings warrant further investigation in larger, well-powered studies to elucidate the potential age-specific effects.

When examining the associations by protein sources, higher dietary plant protein intake, but not total or animal protein, was modestly associated with a lower incidence of sarcopenia. This finding aligns with reports from European (14) and Chinese populations (8), and is supported by recent evidence demonstrating that plant-based proteins can effectively support muscle health when consumed in adequate amounts and appropriate combinations (37). However, given the modest effect size, this association should be interpreted with caution. Plant-based proteins are recognized as being lower in certain essential amino acids (i.e., lysine, leucine) (38, 39), and generally demonstrate a lower capacity to stimulate muscle protein synthesis compared to animal-based proteins (40). Several possible explanations, although these remain speculative, may account for this observation. First, the broader dietary context is critical. Participants consuming higher plant protein may simultaneously maintain adequate total protein and animal protein intake, thereby ensuring sufficient essential amino acid availability. Indeed, evidence indicates that essential amino acid adequacy can be maintained when plant protein constitutes less than 70% of total protein intake (41). In our cohort, 98% of participants had plant protein proportions below this threshold, with a mean proportion of 36%, suggesting that increased plant protein consumption was not accompanied by inadequate essential amino acid intake. Second, plant-based foods contain multiple bioactive compounds beyond protein (e.g., polyphenols, fiber) that may confer independent benefits for muscle health and metabolic function (12). In stratified analyses, the association between plant protein intake and sarcopenia was observed in females and those aged < 65 years, although no statistically significant interactions were detected. These findings were generally consistent with the overall results, and further research is warranted to better understand potential subgroup differences. A key limitation is our focus on overall plant protein intake without distinguishing specific sources (e.g., legumes, cereals, nuts) or dietary practices affecting protein quality. Future research examining specific plant protein sources and dietary patterns would strengthen evidence for targeted sarcopenia prevention strategies.

Chronic inflammation is a recognized contributor to age-related muscle decline (42, 43). In line with this mechanistic link, elevated CRP, WBC, lymphocyte, monocyte, neutrophil, and platelet counts were significantly associated with increased sarcopenia risk in our cohort. Mediation analysis suggested that CRP, WBC, monocyte, and platelet count collectively accounted for a small proportion of the observed association between plant protein intake and sarcopenia incidence, raising the possibility that systemic inflammation may be related to this association. However, the wide confidence intervals (95% CI = 4.8–41.5%) around the mediation estimate suggest considerable uncertainty, and warrant cautious interpretation. This pattern is broadly consistent with longitudinal evidence linking greater plant protein consumption to attenuated systemic inflammation (18). Nevertheless, these findings should be interpreted cautiously, as our macronutrient-level analysis reflects population-level dietary patterns and does not permit inference about the direct effects of isolated plant protein constituents. The observed associations are therefore more plausibly attributable to the broader nutritional matrix of plant-based foods, which are characteristically rich in dietary fiber, polyphenols, carotenoids, vitamin C, and other bioactive phytochemicals (12, 44). These constituents may act synergistically within the food matrix to modulate inflammatory processes associated with muscle proteolysis, thereby supporting muscle integrity in older adults. Further research is needed to better understand the relationships between plant-based protein, inflammatory markers, and sarcopenia.

To our knowledge, this is the first prospective study to explore the joint associations of dietary plant protein intake and genetic risk (MetaPRS) with sarcopenia incidence. Notably, our primary analysis did not support the hypothesis that higher plant protein intake counteracts the elevated sarcopenia risk associated with high genetic susceptibility. In stratified analyses, an inverse association was observed among individuals at higher genetic risk, although no statistically significant interaction was detected. Taken together, these findings provide preliminary insight into the potential interplay between dietary plant protein intake and genetic susceptibility, although further research is warranted to clarify this relationship.

Our study has several strengths. First, the large sample size and prospective design enhance the statistical power and validity of our findings. Second, we constructed a MetaPRS to examine the joint associations of dietary plant protein intake and genetic susceptibility with the incidence of sarcopenia. Furthermore, we explored the mediating role of inflammatory factors in the relationship between plant protein intake and sarcopenia, providing deeper mechanistic insight. Overall, our findings indicate that plant protein intake may be associated with sarcopenia risk and contribute to a better understanding of its underlying mechanisms. Several limitations should be acknowledged. First, dietary intake was assessed using repeated 24-h recalls, which may not fully capture long-term habitual intake or reflect protein quality. This may introduce measurement error and lead to exposure misclassification, potentially attenuating the observed associations. Second, the exclusive use of individuals of white European ancestry in this UK Biobank-based study may limit the applicability of the findings to more ethnically diverse populations. Third, sarcopenia was ascertained at discrete assessment time points, and the assigned event date reflects the timing of measurement rather than the true onset, which may introduce some uncertainty in event timing due to interval censoring and should be considered in interpretation. Finally, the incompleteness of physical examination data and the absence of follow-up information in the UK Biobank may introduce selection bias, as participants included in the final analytic sample may differ systematically from those excluded, which may affect the generalizability of the findings.

## Conclusion

Our findings indicate that higher dietary plant protein intake is modestly associated with a lower incidence of sarcopenia. Mediation analysis suggests that inflammatory biomarkers may be involved in this association. These findings suggest that plant protein intake may be a relevant modifiable dietary factor in relation to sarcopenia risk, and further research is warranted to aid dietary guideline recommendations.

## Data Availability

The original contributions presented in the study are included in the article/Supplementary material, further inquiries can be directed to the corresponding author.
