# Supplementary material for: Dietary protein intake, inflammatory biomarkers, genetic susceptibility, and the incidence of sarcopenia: a prospective population-based study
Source: Front Nutr. 2026 May 4;13:1821758. doi: 10.3389/fnut.2026.1821758 (PMC13180539; doi:10.3389/fnut.2026.1821758)
Supplement: Supplementary file 1 [file Table_1.DOCX]

**Supplemental Materials**

**Dietary protein intake, inflammatory biomarkers, genetic susceptibility, and the incidence of sarcopenia: a prospective population-based study**

Author list: Hongxia Xia^¶^, Rong Xiang^¶^, Xin Song, Yang Qu, Xunying Zhao, Ting Liu, Maoyao Xia, Yangdan Zhong, Zilan Chen, Ye Ju, Yuqi Pang, Zihao Li, Mengyu Fan, Lu Long, Xia Jiang

**Supplementary Figure 1.** Dose-response associations between dietary protein intake and the risk of sarcopenia...............................................................................................2

**Supplementary Table1.** Definition of dietary protein sources in the UKB.................3

**Supplementary Table 2.** Subgroup analyses of the association between dietary protein intake and incident sarcopenia..........................................................................4

**Supplementary Table 3**. Sensitivity analyses for the association between dietary protein intake and sarcopenia incidence.........................................................................5

**Supplementary Table 4.** Association between levels of dietary protein intake, sources of dietary protein intake, and prevalence of sarcopenia in the cross-sectional analysis...........................................................................................................................6

**Supplementary Table 5.** Characteristics of Participants in the Cross-Sectional Study...............................................................................................................................7

**Supplementary Table 6.** Sensitivity analysis using logistic regression models for the association between dietary protein intake and incident sarcopenia..............................8

**Supplementary Table 7.** Association between inflammatory biomarkers and the incidence of sarcopenia..................................................................................................9

**
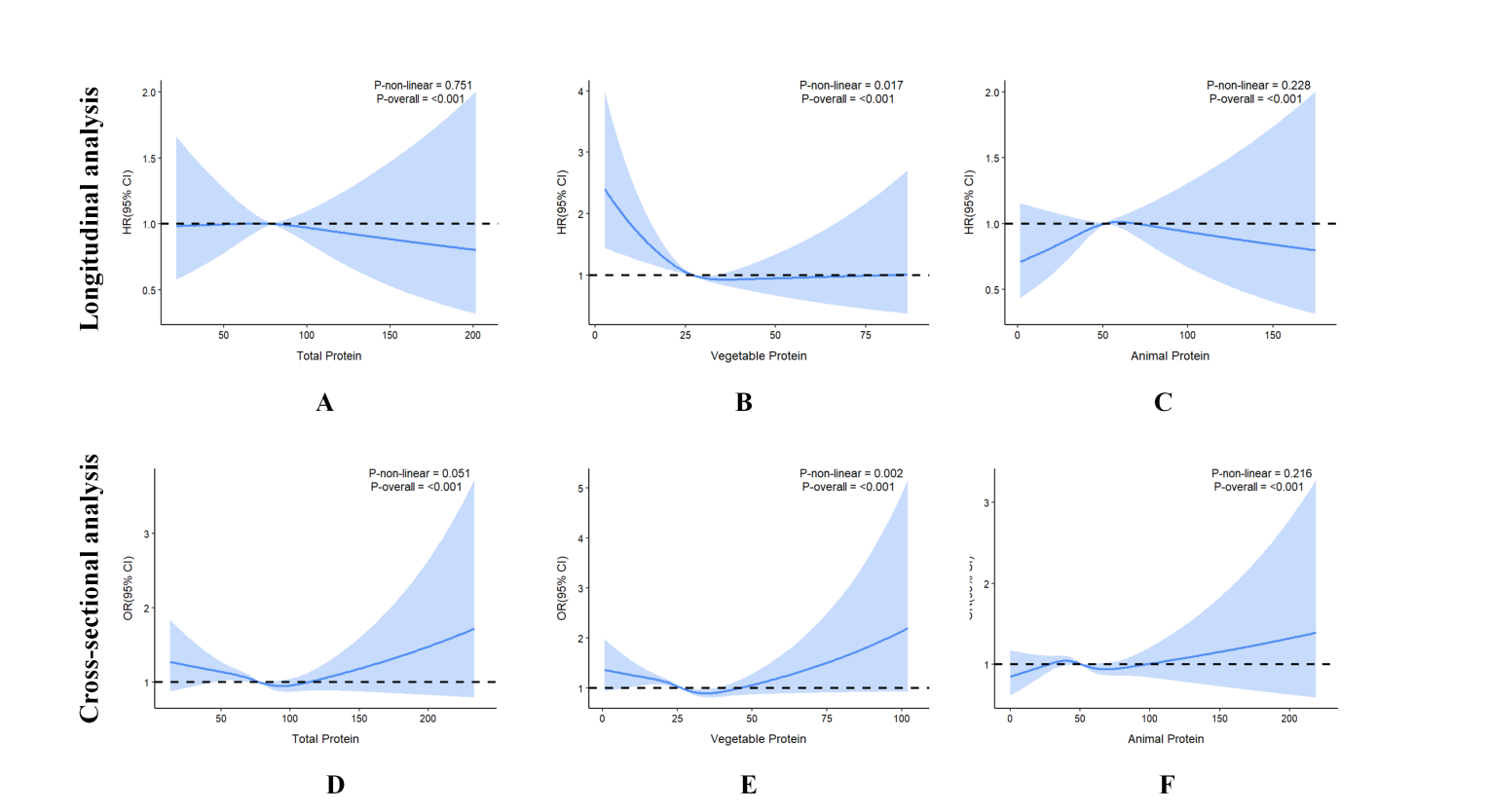
**

**Supplementary Figure 1. Dose-response associations between dietary protein intake and the risk of sarcopenia.**

This figure presents restricted cubic spline (RCS) analyses assessing the associations of continuous dietary protein intake with incidence and prevalence of sarcopenia. Panels A–C illustrate the associations of dietary total, plant, and animal protein intake with the incidence of sarcopenia. Panels D–F depict the corresponding associations with the prevalence of sarcopenia.

All analyses were adjusted according to Model 3 covariates: age, sex, education level, Townsend deprivation index, body mass index, total physical activity level, smoking, alcohol consumption, hypertension, hyperlipidemia, diabetes, and energy intake.

| **Supplementary Table1. Definition of dietary protein sources in the UKB** | | |
| --- | --- | --- |
| Protein sources | Food items | ID |
| Whole grains | Biscuit cereal,  Bran cereal,  Oat cereal (no sugar),  Oat cereal (sugar),  Muesli,  Whole meal pasta, brown rice and other whole grains | 26075,  26076,  26077,  26078,  26105,  26114 |
| Nuts | Unsalted nuts and seeds, Salted nuts and seeds | 26107,  26108 |
| Legumes | Legumes and pulses | 26101 |
| Red meat | Beef,  Lamb,  Pork | 26066,  26100,  26117 |
| Processed meat | Processed meat | 26122 |
| Poultry | Poultry | 26121 |
| Egg and egg dishes | Egg and egg dishes | 26088 |
| Oily fish | Oily fish | 26109 |
| Non-oily fish | Breaded/battered fish,  White fish and tinned tuna | 26070,  26149 |
| Cheese | High fat cheese,  Medium and low fat cheese | 26099,  26103 |
| Milk | Semi skimmed milk, Skimmed milk and cholesterol-lowering milk, Whole milk | 26131,  26133,  26150 |
| Yogurt | Full fat yogurt,  Low fat yogurt | 26096,  26102 |

| **Supplementary Table 2. Subgroup analyses of the association between dietary protein intake and incident sarcopenia.** | | | | | |
| --- | --- | --- | --- | --- | --- |
| **Subgroup** | **Quartiles of Dietary Protein Intake Grouped (g/day)** | | | | ***P* for interaction** |
|  | **Q1** | **Q2** | **Q3** | **Q4** |  |
| **Total protein** |  |  |  |  |  |
| Age |  |  |  |  | 0.362 |
| ≥65 | 1.00 [Ref] | 1.12 (0.79,1.59) | 1.13 (0.77,1.67) | 1.27 (0.82,1.97) |  |
| <65 | 1.00 [Ref] | **0.74 (0.55,0.99)** | 0.75 (0.55,1.02) | 0.79 (0.56,1.13) |  |
| Sex |  |  |  |  | 0.476 |
| Female | 1.00 [Ref] | 0.95 (0.72,1.26) | 0.98 (0.73,1.32) | 1.16 (0.84,1.61) |  |
| Male | 1.00 [Ref] | 1.27 (0.86,1.87) | 0.90 (0.58,1.42) | 0.87 (0.52,1.43) |  |
| **Plant protein** |  |  |  |  |  |
| Age |  |  |  |  | 0.673 |
| ≥65 | 1.00 [Ref] | 1.01 (0.73,1.40) | 0.74 (0.51,1.09) | 0.87 (0.57,1.34) |  |
| <65 | 1.00 [Ref] | **0.74 (0.56,0.97)** | 0.85 (0.63,1.15) | **0.71 (0.50,0.99)** |  |
| Sex |  |  |  |  | 0.761 |
| Female | 1.00 [Ref] | 0.82 (0.63,1.07) | **0.74 (0.56,0.99)** | **0.73 (0.53,0.99)** |  |
| Male | 1.00 [Ref] | 0.82 (0.56,1.20) | 0.82 (0.54,1.25) | 0.77 (0.49,1.23) |  |
| **Animal protein** |  |  |  |  |  |
| Age |  |  |  |  | 0.232 |
| ≥65 | 1.00 [Ref] | 1.25 (0.87,1.78) | 1.33 (0.93,1.91) | 1.22 (0.82,1.80) |  |
| <65 | 1.00 [Ref] | 1.07 (0.80,1.43) | 0.98 (0.72,1.32) | 1.06 (0.77,1.45) |  |
| Sex |  |  |  |  | 0.533 |
| Female | 1.00 [Ref] | 1.03 (0.78,1.36) | 1.04 (0.78,1.38) | 1.14 (0.85,1.52) |  |
| Male | 1.00 [Ref] | 1.17 (0.79,1.73) | 1.01 (0.66,1.52) | 0.96 (0.62,1.49) |  |
| Data are shown as HR (95%CI). Abbreviation: HR, Hazard Ratio; CI, confidence interval; Q, quartile. All analyses were adjusted according to Model 3 covariates: age, sex, education level, Townsend deprivation index, body mass index, total physical activity level, smoking, alcohol consumption, hypertension, hyperlipidemia, diabetes, and energy intake. P for interaction values were derived from tests of multiplicative interaction between quartiles of dietary protein intake and each subgroup variable. | | | | | |

| **Supplementary Table 3. Sensitivity analyses for the association between dietary protein intake and sarcopenia incidence.** | | | | | | | | |
| --- | --- | --- | --- | --- | --- | --- | --- | --- |
| **Protein sources** |  | **Sensitivity analysis 1** | | |  | **Sensitivity analysis 2** | | |
|  |  | **Levels (g/day)** | ***HR* (95%*CI*)** | ***P*-value** |  | **Levels (g/day)** | ***HR* (95%*CI*)** | ***P*-value** |
| **Total protein** |  |  |  |  |  |  |  |  |
| Q1, lowest |  | ≤66.88 | 1.00 [Ref] | |  | ≤66.87 | 1.00 [Ref] | |
| Q2 |  | 66.88-79.26 | 0.83 (0.67-1.04) | 0.113 |  | 66.87-78.36 | 0.79 (0.63-0.99) | **0.048** |
| Q3 |  | 79.26-92.78 | 0.85 (0.67-1.09) | 0.199 |  | 78.36-90.49 | 0.78 (0.61-0.99) | **0.049** |
| Q4, highest |  | ≥92.78 | 0.95 (0.72-1.25) | 0.699 |  | ≥90.49 | 0.95 (0.72-1.25) | 0.712 |
| **Plant protein** |  |  |  |  |  |  |  |  |
| Q1, lowest |  | ≤22.26 | 1.00 [Ref] | |  | ≤22.22 | 1.00 [Ref] | |
| Q2 |  | 22.26-27.32 | 0.83 (0.68-1.03) | 0.088 |  | 22.22-26.92 | 0.76 (0.61-0.96) | **0.018** |
| Q3 |  | 27.32-33.12 | 0.75 (0.59-0.95) | **0.019** |  | 26.92-32.08 | 0.80 (0.63-1.02) | 0.075 |
| Q4, highest |  | ≥33.12 | 0.76 (0.58-0.99) | **0.043** |  | ≥32.08 | 0.75 (0.58-0.99) | **0.042** |
| **Animal protein** |  |  |  |  |  |  |  |  |
| Q1, lowest |  | ≤40.18 | 1.00 [Ref] | |  | ≤41.05 | 1.00 [Ref] | |
| Q2 |  | 40.18-51.43 | 1.12 (0.90-1.41) | 0.315 |  | 41.05-51.32 | 1.06 (0.84-1.34) | 0.615 |
| Q3 |  | 51.43-62.81 | 1.10 (0.87-1.39) | 0.416 |  | 51.32-61.75 | 1.09 (0.86-1.38) | 0.494 |
| Q4, highest |  | ≥62.81 | 1.12 (0.88-1.43) | 0.358 |  | ≥61.75 | 1.10 (0.86-1.42) | 0.438 |
| Abbreviation: HR, Hazard Ratio; CI, confidence interval; Q, quartile. Sensitivity analysis 1 excluded participants with missing covariates data; Sensitivity analysis 2 excluded participants with protein intake below the 2.5th percentile or above the 97.5th percentile. The sensitivity analyses were adjusted according to Model 3 covariates: age, sex, education level, Townsend deprivation index, body mass index, total physical activity level, smoking, alcohol consumption, hypertension, hyperlipidemia, diabetes, and energy intake. | | | | | | | | |

| **Supplementary Table 4. Association between levels of dietary protein intake, sources of dietary protein intake, and prevalence of sarcopenia in the cross-sectional analysis.** | | | | | | | | | | | |
| --- | --- | --- | --- | --- | --- | --- | --- | --- | --- | --- | --- |
| **Protein sources** |  | **Protein intake(g/day)** |  | **Model 1** | |  | **Model 2** | |  | **Model 3 (main model)** | |
|  |  |  |  | ***OR* (95%*CI*)** | ***P*-value** |  | ***OR* (95%*CI*)** | ***P*-value** |  | ***OR* (95%*CI*)** | ***P*-value** |
| **Total protein** |  |  |  |  |  |  |  |  |  |  |  |
| Q1, lowest |  | ≤65.14 |  | 1.00 [Ref] | |  | 1.00 [Ref] | |  | 1.00 [Ref] | |
| Q2 |  | 65.14-78.00 |  | 0.89 (0.81-0.98) | **0.016** |  | 0.96 (0.86-1.08) | 0.538 |  | 1.05 (0.93-1.19) | 0.446 |
| Q3 |  | 78.00-91.92 |  | 0.74 (0.67-0.82) | **<0.001** |  | 0.80 (0.71-0.91) | **<0.001** |  | 0.93 (0.81-1.07) | 0.316 |
| Q4, highest |  | ≥91.92 |  | 0.86 (0.78-0.95) | **0.004** |  | 0.69 (0.61-0.79) | **<0.001** |  | 0.89 (0.75-1.04) | 0.142 |
| **Plant protein** |  |  |  |  |  |  |  |  |  |  |  |
| Q1, lowest |  | ≤21.41 |  | 1.00 [Ref] | |  | 1.00 [Ref] | |  | 1.00 [Ref] | |
| Q2 |  | 21.41-26.61 |  | 0.72 (0.66-0.79) | **<0.001** |  | 0.79 (0.71-0.89) | **<0.001** |  | 0.85 (0.75-0.95) | **0.006** |
| Q3 |  | 26.61-32.63 |  | 0.59 (0.53-0.65) | **<0.001** |  | 0.67 (0.59-0.75) | **<0.001** |  | 0.75 (0.65-0.86) | **<0.001** |
| Q4, highest |  | ≥32.63 |  | 0.59 (0.53-0.66) | **<0.001** |  | 0.66 (0.58-0.75) | **<0.001** |  | 0.80 (0.68-0.93) | **0.005** |
| **Animal protein** |  |  |  |  |  |  |  |  |  |  |  |
| Q1, lowest |  | ≤39.34 |  | 1.00 [Ref] | |  | 1.00 [Ref] | |  | 1.00 [Ref] | |
| Q2 |  | 39.34-50.85 |  | 0.95 (0.86-1.05) | 0.280 |  | 0.92 (0.81-1.04) | 0.194 |  | 0.97 (0.86-1.10) | 0.648 |
| Q3 |  | 50.85-62.52 |  | 0.94 (0.85-1.04) | 0.266 |  | 0.93 (0.82-1.05) | 0.245 |  | 1.04 (0.91-1.18) | 0.573 |
| Q4, highest |  | ≥62.52 |  | 1.00 (0.91-1.11) | 0.928 |  | 0.76 (0.67-0.86) | **<0.001** |  | 0.92 (0.80-1.06) | 0.235 |
| Abbreviation: OR, Odds Ratio; CI, confidence interval; Q, quartile. Model 1: adjusted for age and sex. Model 2: additionally adjusted for education level, Townsend deprivation index, body mass index, total physical activity level, smoking status and alcohol consumption on top of Model 1. Model 3: additionally adjusted for hypertension, hyperlipidemia, diabetes and energy intake on top of Model 2. | | | | | | | | | | | |

| **Supplementary Table 5. Characteristics of Participants in the Cross-Sectional Study.** | | | |
| --- | --- | --- | --- |
| **Characteristics** | **Without Sarcopenia**  **(N=188,961)** | **With Sarcopenia**  **(N=3,037)** | ***P*-value** |
| **Basic characteristics** | | | |
| Age, years | 58.47±7.95 | 63.30±6.26 | <0.001 |
| Sex |  |  | <0.001 |
| Female | 105,348 (55.75) | 2,368 (77.97) |  |
| male | 83,613 (44.25) | 669 (22.03) |  |
| College or university degree | 110,297 (58.37) | 1,167 (38.23) | <0.001 |
| Body mass index (kg/m²) | 26.81±4.55 | 32.51±5.65 | <0.001 |
| Townsend deprivation index | -1.70±2.79 | -1.04±3.09 | <0.001 |
| High physical activity level | 61,765 (32.69) | 565 (18.60) | <0.001 |
| Sleep 7-8 h/day | 135,105 (71.50) | 1,813 (59.70) | <0.001 |
| Smoking status |  |  | <0.001 |
| Never | 106,358 (56.29) | 1,624 (53.47) |  |
| Previous | 67,960 (33.97) | 1,211 (39.87) |  |
| Current | 14,262 (7.55) | 187 (6.16) |  |
| Missing | 381 (2.19) | 15 (0.50) |  |
| Alcohol consumption |  |  | <0.001 |
| Never | 4,823 (2.55) | 178 (5.86) |  |
| Previous | 5,395 (2.86) | 199 (6.55) |  |
| Current | 178,669 (94.55) | 2,659 (87.55) |  |
| Missing | 74 (0.04) | 1 (0.04) |  |
| Hypertension | 15,842 (8.38) | 714 (23.51) | <0.001 |
| Hyperlipidemia | 6,460 (3.42) | 256 (8.43) | <0.001 |
| Diabetes | 2,956 (1.56) | 205 (6.75) | <0.001 |
| **Nutrients intake** |  |  |  |
| Energy intake (kcal/day) | 2,019.39±482.47 | 1,872.76±482.21 | <0.001 |
| Carbohydrate intake (g/day) | 248.89±68.07 | 235.51±68.01 | <0.001 |
| Fat intake (g/day) | 71.22±24.54 | 66.08±24.66 | <0.001 |
| Total protein (g/day) | 79.34±21.38 | 76.63±22.62 | <0.001 |
| Plant protein (g/day) | 27.66±9.30 | 25.24±8.91 | <0.001 |
| Animal protein (g/day) | 51.68±19.50 | 51.39±19.75 | 0.061 |
| **Inflammatory biomarkers** * |  |  |  |
| CRP (mg/L) | 0.21±1.04 | 1.11±0.98 | <0.001 |
| WBC (10^9 cells/L) | 1.88±0.25 | 1.98±0.26 | <0.001 |
| Lymphocyte count (10^9 cells/L) | 0.60±0.32 | 0.69±0.35 | <0.001 |
| Monocyte count (10^9 cells/L) | -0.82±0.37 | -0.76±0.41 | <0.001 |
| Neutrophil count (10^9 cells/L) | 1.37±0.33 | 1.47±0.35 | <0.001 |
| Platelet count (10^9 cells/L) | 5.49±0.24 | 5.55±0.27 | <0.001 |
| Data are presented as mean±standard deviation) or number (%). Abbreviations: WBC, white blood cell; CRP, C-reactive protein. *The levels of inflammatory biomarkers were natural log-transformed before analysis. | | | |

| **Supplementary Table 6. Sensitivity analysis using logistic regression models for the association between dietary protein intake and incident sarcopenia.** | | | | | | | | | | | |
| --- | --- | --- | --- | --- | --- | --- | --- | --- | --- | --- | --- |
| **Protein sources** |  | **Protein intake(g/day)** |  | **Model 1** | |  | **Model 2** | |  | **Model 3 (main model)** | |
|  |  |  |  | ***OR* (95%*CI*)** | ***P*-value** |  | ***OR* (95%*CI*)** | ***P*-value** |  | ***OR* (95%*CI*)** | ***P*-value** |
| **Total protein** |  |  |  |  |  |  |  |  |  |  |  |
| Q1, lowest |  | ≤66.72 |  | 1.00 [Ref] | |  | 1.00 [Ref] | |  | 1.00 [Ref] | |
| Q2 |  | 66.72-79.13 |  | 0.88 (0.72-1.06) | 0.182 |  | 0.84 (0.67-1.05) | 0.129 |  | 0.87 (0.69-1.09) | 0.224 |
| Q3 |  | 79.13-92.60 |  | 0.90 (0.74-1.10) | 0.297 |  | 0.82 (0.66-1.03) | 0.092 |  | 0.87 (0.67-1.11) | 0.266 |
| Q4, highest |  | ≥92.60 |  | 1.14 (0.94-1.39) | 0.176 |  | 0.88 (0.71-1.10) | 0.274 |  | 0.96 (0.72-1.28) | 0.783 |
| **Plant protein** |  |  |  |  |  |  |  |  |  |  |  |
| Q1, lowest |  | ≤22.20 |  | 1.00 [Ref] | |  | 1.00 [Ref] | |  | 1.00 [Ref] | |
| Q2 |  | 22.20-27.22 |  | 0.73 (0.61-0.88) | **0.001** |  | 0.77 (0.63-0.96) | **0.018** |  | 0.77 (0.61-0.96) | **0.019** |
| Q3 |  | 27.22-32.96 |  | 0.71 (0.58-0.85) | **<0.001** |  | 0.76 (0.61-0.94) | **0.013** |  | 0.74 (0.58-0.95) | **0.019** |
| Q4, highest |  | ≥32.96 |  | 0.69 (0.56-0.84) | **<0.001** |  | 0.74 (0.59-0.93) | **0.010** |  | 0.72 (0.55-0.96) | **0.024** |
| **Animal protein** |  |  |  |  |  |  |  |  |  |  |  |
| Q1, lowest |  | ≤40.17 |  | 1.00 [Ref] | |  | 1.00 [Ref] | |  | 1.00 [Ref] | |
| Q2 |  | 40.17-51.36 |  | 1.12 (0.91-1.37) | 0.272 |  | 1.10 (0.87-1.39) | 0.413 |  | 1.13 (0.90-1.43) | 0.297 |
| Q3 |  | 51.36-62.83 |  | 1.17 (0.96,1.44) | 0.126 |  | 1.07 (0.85-1.35) | 0.560 |  | 1.12 (0.88-1.43) | 0.345 |
| Q4, highest |  | ≥62.83 |  | 1.39 (1.14-1.70) | **0.001** |  | 1.04 (0.83-1.32) | 0.724 |  | 1.13 (0.88-1.46) | 0.334 |
| Abbreviation: OR, Odds Ratio; CI, confidence interval; Q, quartile. Model 1: adjusted for age and sex. Model 2: additionally adjusted for education level, Townsend deprivation index, body mass index, total physical activity level, smoking status and alcohol consumption on top of Model 1. Model 3: additionally adjusted for hypertension, hyperlipidemia, diabetes and energy intake on top of Model 2. | | | | | | | | | | | |

| **Supplementary Table 7. Association between inflammatory biomarkers and the incidence of sarcopenia.** | | |
| --- | --- | --- |
| **Inflammatory biomarkers*** | ***HR* (95%*CI*)** | ***P*-value** |
| CRP (mg/L) | 1.23 (1.13,1.35) | **<0.001** |
| WBC (10^9 cells/L) | 1.75 (1.26,2.42) | **<0.001** |
| Lymphocyte count (10^9 cells/L) | 1.31 (1.02,1.68) | **0.037** |
| Monocyte count (10^9 cells/L) | 1.35 (1.08,1.69) | **0.009** |
| Neutrophil count (10^9 cells/L) | 1.42 (1.10,1.84) | **0.008** |
| Platelet count (10^9 cells/L) | 2.30 (1.61,3.28) | **<0.001** |
| Abbreviations: WBC, white blood cell; CRP, C-reactive protein; HR, Hazard Ratio; CI, confidence interval. *The levels of inflammatory biomarkers were natural log-transformed before analysis. The analyses were adjusted according to Model 3 covariates: age, sex, education level, Townsend deprivation index, body mass index, total physical activity level, smoking, alcohol consumption, hypertension, hyperlipidemia, diabetes, and energy intake. | | |
